# Sacubitril/Valsartan contributes to improving the diabetic kidney disease and regulating the gut microbiota in mice

**DOI:** 10.3389/fendo.2022.1034818

**Published:** 2022-12-16

**Authors:** Peipei Wang, Ruixue Guo, Xiwen Bai, Wen Cui, Yiding Zhang, Huangmin Li, Jin Shang, Zhanzheng Zhao

**Affiliations:** ^1^ Department of Nephrology, The First Affiliated Hospital of Zhengzhou University, Zhengzhou, China; ^2^ Zhengzhou University, Zhengzhou, China; ^3^ Nanchang University Queen Mary School, Nanchang, China; ^4^ Laboratory Animal Platform of Academy of Medical Sciences, Zhengzhou University, Zhengzhou, China; ^5^ Nephropathy Laboratory, The First Affiliated Hospital of Zhengzhou University, Zhengzhou, China

**Keywords:** sacubitril/valsartan, diabetic kidney disease, gut microbiota, metabolome, mice

## Abstract

**Background:**

Diabetic kidney disease (DKD), as a serious microvascular complication of diabetes, has limted treatment options. It is reported that the Sacubitril/Valsartan (Sac/Val) can improve kidney function, and the disordered gut microbiota and part of its metabolites are related to the development of DKD. Therefore, we aim to explore whether the effect of Sac/Val on DKD is associated with the gut microbiota and related plasma metabolic profiles.

**Methods:**

Male C57BL/6J mice were randomly divided into 3 groups: Con group (n = 5), DKD group (n = 6), and Sac/Val group (n = 6) . Sac/Val group was treated with Sac/Val solution. The intervention was given once every 2 days for 6 weeks. We measured the blood glucose and urine protein level of mice at different times. We then collected samples at the end of experiment for the 16s rRNA gene sequencing analysis and the untargeted plasma metabonomic analysis.

**Results:**

We found that the plasma creatinine concentration of DKD-group mice was significantly higher than that of Con-group mice, whereas it was reduced after the Sac/Val treatment. Compared with DKD mice, Sac/Val treatment could decrease the expression of indicators related to EndMT and renal fibrosis like vimentin, collagen IV and fibronectin in kidney. According to the criteria of LDA ≥ 2.5 and p<0.05, LefSe analysis of gut microbiota identified 13 biomarkers in Con group, and 33 biomarkers in DKD group, mainly including *Prevotella, Escherichia_Shigella* and *Christensenellaceae_R_7_group*, etc. For the Sac/Val group, there were 21 biomarkers, such as *Bacteroides, Rikenellaceae_RC9_gut_group, Parabacteroides, Lactobacillus*, etc. Plasma metabolomics analysis identified a total of 648 metabolites, and 167 important differential metabolites were screened among groups. KEGG pathway of tryptophan metabolism: M and bile secretion: OS had the highest significance of enrichment.

**Conclusions:**

Sac/Val improves the renal function of DKD mice by inhibiting renal fibrosis. This drug can also regulate gut microbiota in DKD mice.

## Introduction

As one of the serious microvascular complications of diabetic mellitus (DM), diabetic kidney disease (DKD) is becoming a common disease threatening health. Overall, about one third of patients with diabetic will gradually get the DKD (1) and eventually suffer from the end-stage renal disease. However, the pathogenesis of DKD is quite complex, which involves the chronic low-grade inflammation, accumulation of advanced glycation end products, and oxidative stress (2, 3). Currently, there is no specific treatment besides general management like controlling blood glucose, lipid, blood pressure and lifestyle adjustment (4).

To date, considerable studies have linked the onset and treatment of DKD with gut microbiota. On the one hand, due to the change of the physical and chemical properties of the intestinal microenvironment in DKD state, the intestinal microbiota of patients is disturbed (5–7). These changes include the predicted function of gut microbiota, a significant reduction in the relative abundance of the butyrate-producing bacteria, SCFAs-producing bacteria and potential probiotics (5, 7), an increase in genus *Escherichia*, *Citrobacter* and *Klebsiella*, and a decrease in of *Roseburia* (6). Abnormal gut microbiota can damage the kidneys by activating the intrarenal renin–angiotensin system and producing harmful metabolites (8–10). On the other hand, the treatment targeting intestinal flora can also improve DKD (11, 12). As we know, intestinal bacteria can produce a large number of metabolites in the intestinal tract. Such metabolites penetrate into the blood and will have different effects on the host. With the disorder of intestinal flora, the plasma metabolic spectrum of individuals with DKD will be disturbed to a certain extent (13, 14). The accumulation of harmful metabolites such as the indoxyl sulfate, phenyl sulfate, and p-cresyl sulfate, may damage the kidney by activating the immune system or other signal pathways (9, 15).

Sac/Val is the first angiotensin receptor-neprilysin inhibitor drug, containing the valsartan and sacubitril as the angiotensin-receptor blocker and neprilysin inhibitor, respectively (16). It contributes to the treatment of the chronic heart failure with reduced ejection fraction (HFrEF) (17, 18). In Sprague-Dawley (SD) rats with cardiorenal syndrome (CRS), Sac/Val was found to improve the heart and renal functions mainly by inhibiting the oxidative stress, fibrosis, and other aspects (19). It can also reduce the blood glucose, glycated hemoglobin (18, 20). Especially, Sac/Val can enhance the peripheral insulin sensitivity for hypertensive patients with obesity without changing the adipose tissue metabolic phenotype (21). For patients with chronic HFrEF and type 2 diabetes (T2D), researchers observed an improved renal function profile when Sac/Val was added to empagliflozin (22). However, no in-depth study has been conducted on exactly how Sac/Val benefits the kidneys in DKD. Given that the relationship between the Sac/Val treatment for DKD and the gut microbiota or plasma metabolic profile remains unclear, we aim to clarify the effects of Sac/Val on the renal damage, intestinal flora and plasma metabolism in DKD through mice experiments.

## Methods

### Animals

We purchased 6-week-old specific pathogen-free (SPF) male C57BL/6 J mice from the Experimental Animal Center of Zhengzhou University and raised them in SPF conditions. They were alternately exposed to light and darkness for 12 hours in turn at the temperature of 22-24°C, with adequate food and water. We received an approval from the Ethical Committee of Experimental Animal Care of First Affiliated Hospital of Zhengzhou University.

### Different interventions of mice in each group

In this experiment, we used Entresto (En), a widely used Sac/Val, for animal treatment. We randomly divided the mice into the normal control (Con, n = 5), DKD model (DKD, n = 6), and En treatment (En, n = 6) groups. After 7-day adaptive feeding, we capture the baseline values of random blood glucose (RBG) and body weight of mice. Subsequently, the mice were injected intraperitoneally with streptozotocin(STZ) at 55 mg/kg for 7 consecutive days and were given high-fat diet (HFD), except for the mice in the Con group. Seven days after the final injection, mice with RBG equal to or higher than 16.7mmol/l were regarded as successful diabetic models for follow-up studies.

Mice in En group were treated with Sac/Val (60mg/kg body weight). The gavage was once every other day for 6 weeks. During treatment, urine samples were collected, and RBG was measured at different times. At the end of experiment, we measured body weight and collected fecal samples. Then the mice were anesthetized, plasma was collected. After the kidneys were perfused with normal saline, they were stored in formaldehyde tissue fixative solution and at -80°C separately for further analysis. Urine, feces and plasma samples were stored in –80°C refrigerators after collection.

### Histopathological analysis of kidney tissue

Kidney tissue was embedded in paraffin and sectioned. We then observed the pathological changes of renal tissue by operations regarding the Hematoxylin-eosin (HE), Masson and Periodic Acid-Schiff (PAS) staining. Finally, immunohistochemical (IHC) staining was performed to observe expression level of proteins.

### Quantitative detection of urinary protein and plasma creatinine

Detection of mouse urinary protein, (T/Cr, Urinary total protein / Urinary creatinine) was completed in the Nephropathy Laboratory of the First Affiliated Hospital of Zhengzhou University. Plasma creatinine was measured by sarcosine oxidase method according to the instructions of kit (Jiancheng Bioengineering Research Institute, Nanjing, China).

### Gut microbial DNA extraction and PCR amplification

Cell membrane and nuclear membrane were lysed by chemical lysis and Beads-Beating methods. Then, using the E.Z.N.A.Stool DNA Kit (Omega Bio-tek, Inc., GA), the DNA of each fecal sample was extracted. The extracted DNA, as a template, was applied to amplify V3 ~ V4 region of 16s ribosomal RNA (16s rRNA) gene.

### 16s rRNA gene sequencing and analysis

The DNA products of different samples were mixed in the same proportion and sequenced with Miseq platform (Illumina Inc., USA). USEARCH was used to clean raw data. Additionally, the UPARSE (Version 7.1) was employed to remove chimeric sequences sort, and the classification of operational taxonomic units (OTUs) was conducted by 97% similarity, followed by the analysis of the phylogenetic membership of each 16S rRNA gene sequence conducted by the RDP classifier and database (Release 11) with a confidence threshold of 70%.

The Mann-Whitney U test and Kruskal-Wallis test were employed for comparison analysis. The weighted UniFrac and principal coordinate analysis (PCoA) plots were obtained by QIIME software. The β diversity across samples was assessed by non-metric multidimensional scaling (NMDS) analysis. We used the PERMANOVA to examine whether the groups were statistically significant and also used the linear discriminant analysis (LDA) effect size (LEfSe) to identify taxa with differential abundance among groups. Bar plots and PCoA plots were drawed with R software. Functional prediction analysis of gut microbiome was performed using PICRUSt2 function prediction tool combined with KEGG database query.

### Untargeted plasma metabolomics determination and analysis

The plasma samples preparation and LC-MS analysis were performed according to the previous description (23). As a metabolomics processing software, Progenesis QI (WatersCorporation, Milford, USA) was used to analyze the LC-MS raw data, by which a data matrix of retention time, mass charge ratio, and peak intensity was reaped. To obtain the metabolite information, the MS and MS/MS mass spectrometry information was matched with the metabolism public databases named HMDB and metlin.

By the ropls package rooted in R software, we conducted the principal component analysis (PCA) and partial least-squares discrimination analysis (PLS-DA). Permutation testing was performed to evaluate the accuracy of the PLS-DA model. The selection of significantly different metabolites was determined based on the variable important in projection (VIP > 1) obtained by PLS-DA model and the p value of Student’s t test (*p* < 0.05). By the metabolic pathway annotation in KEGG database, we obtained the pathways involved in differential metabolites and employed Python to analyze their enrichment.

### Data analysis

We used GraphPad Prism 8 software for statistical analysis. Comparisons of RBG and T/Cr at different time points among groups were performed by two-way ANOVA multiple comparisons. One-way ANOVA multiple comparisons was used for statistical analysis of ratio of right kidney weight/ body weight and plasma creatinine. *p* < 0.05 was considered statistically significant.

## Results

### Sac/Val treatment improved renal function and alleviated pathological damage of DKD mice

**Figure 1:**
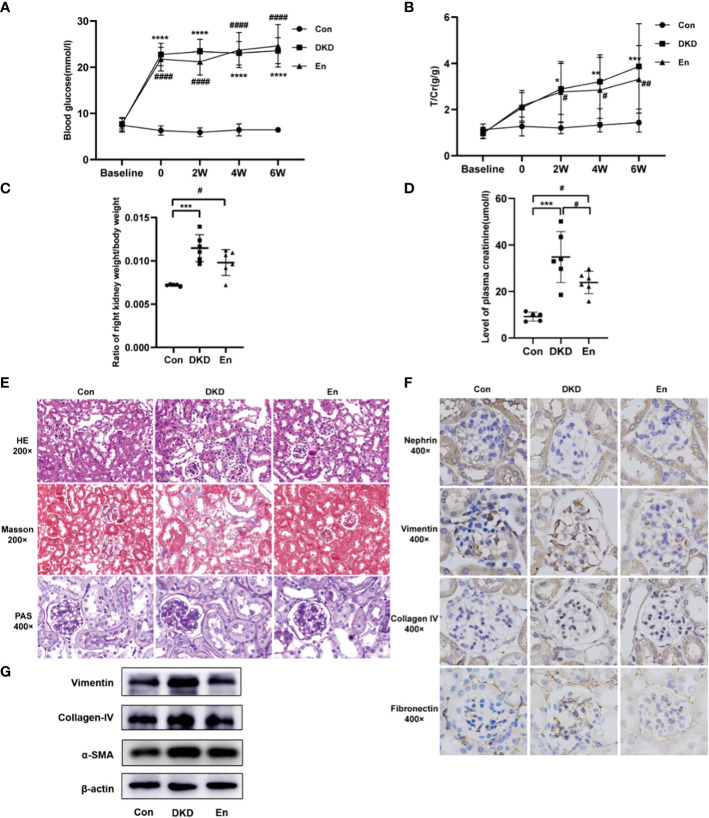
Analysis of general, laboratory and pathological indexes. **(A, B)** Line graphs of mouse RBG and T/Cr at different time points. **(C)** Scatter plot of ratio of right kidney weight/body weight. **(D)** Scatter plot of plasma creatinine. **(E)** HE/Masson/PAS staining of mouse kidney tissue in each group. **(F)** IHC staining of nephrin, vimentin, collagen IV, fibronectin expression in kidney tissue. **(G)** Expression levels of vimentin, collagen IV, α-SMA in kidney tissue by Western blot analysis. *: DKD VS Con, ^#^: En VS Con and En VS DKD. * and ^#^ *p* < 0.05, ** and ^##^ *p* < 0.01, ***p < 0.001, **** and ^####^ *p* < 0.0001.

Taking the DKD group as reference, we found that Sac/Val treatment had no significant effect on RBG and urine protein in mice (*p*> 0.05) (**Figures 1A, B**). We found an increase of the ratio of right kidney weight/ body weight (*p*< 0.05) (**Figure 1C**). After Sac/Val treatment, the ratio slightly decreased compared with DKD group, but it did not reach a significant level (*p*> 0.05). Plasma creatinine concentration is important indicator of renal function, we compared its differences among the three groups. **Figure 1D** showed that the DKD group had significantly higher creatinine level than the Con group and En group (*p*< 0.05). Thus, Sac/Val treatment improved the renal function.

Sac/Val treatment alleviated the pathological damage of DKD kidney. DKD mice showed obvious focal tubular atrophy, renal interstitial fibrosis, increased fibroblasts, and inflammatory cell infiltration. While Sac/Val significantly attenuated these lesions and glycogen deposition (**Figure 1E**). Compared with Con mice, the indicators related to renal fibrosis and endothelial-to-mesenchymal transition (EndMT) like collagen IV, vimentin and fibronectin were significantly increased in the DKD group, but decreased in the En group (**Figure 1F**). The Western blot results of renal tissue further verified the Masson and IHC staining results of collagen IV and vimentin. Moreover, α-SMA, another protein involved in renal fibrosis, decreased in Sac/Val treatment mice (**Figure 1G**). Overall, these results showed that intervention with En can improve renal fibrosis of DKD.

### Characteristics of gut microbiome in three groups of mice

**Figure 2:**
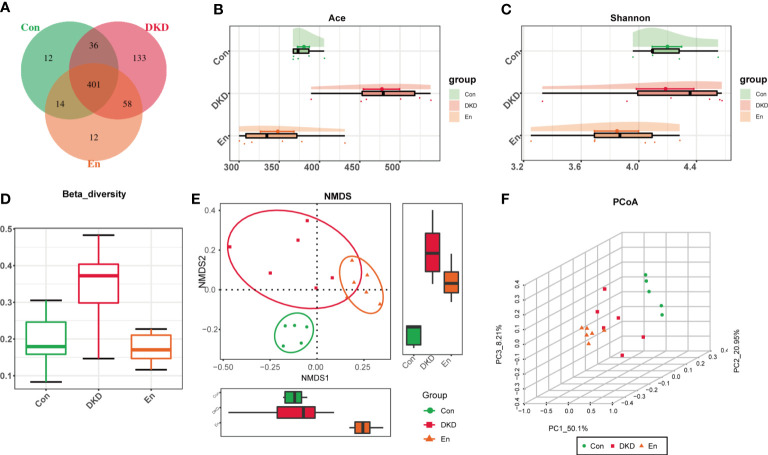
Features of gut microbiota. **(A)** Venn diagram of OTU distribution. **(B)** Ace index: estimate the number of OTUs in fecal samples. **(C)** Cloud plot of Shannon index. **(D)** Box diagram of *β* diversity based on weighted-Unifrac distance. **(E)** NMDS analysis based on Bray-Curtis algorithm. **(F)** 3D plot of PCoA analysis based on weighted-UniFrac distance. PC1, PC2 and PC3 represent the first principal coordinate, the second principal coordinate and the third principal coordinate, respectively.

In the gut microbiome of the three groups, 666 OTUs were identified, of which 401 were common among the three groups and 12 were unique to the En group (**Figure 2A**). DKD group had the largest number of OTUs, followed by Con group, and En group had the least number (**Figure 2B**). Shannon index indicated that the α diversity of gut microbiota had no statistical difference among the three groups (**Figure 2C**). The gut microbiome of DKD group had the highest β diversity (**Figure 2D**). NMDS analysis and PCoA showed that the gut microbiome of the three groups were separated from each other, suggesting a distinct clustering of gut microbiota composition for each group. (**Figures 2E, F**).

### Comparison of gut microbiome composition in each group

**Figure 3:**
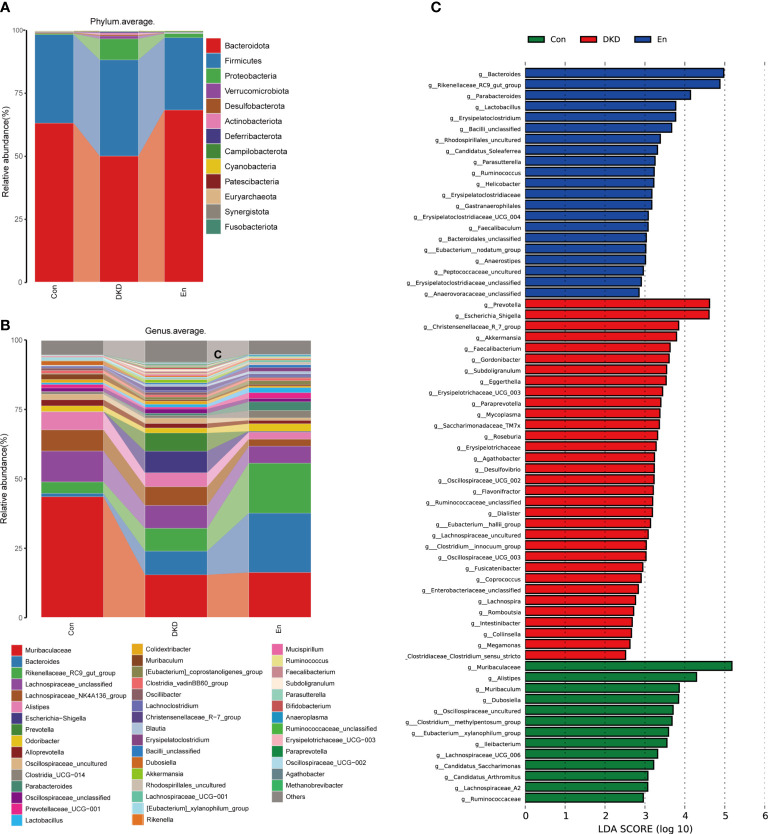
Analysis of gut microbiota composition and biomarkers in different groups. **(A, B)** Bar plots of the composition of intestinal flora at the phylum and genus levels. **(C)** LefSe analysis of microbial profiles in each groups at the genus level (LDA ≥ 2.5).

Next, we focused on the differences of gut microbiome at different levels in each group. At the phylum level, *Bacteroidetes*, *Firmicutes* and *Proteobacteria* were the three most dominant phyla. Although the difference did not reach statistical significance, the relative abundance of *Bacteroidetes* decreased in DKD group. However, this change was reversed in En group. The ratios of *Firmicutes* to *Bacteroidota* were 0.56 and 0.76 in Con and DKD group, respectively. Such ratio decreased in En group, which was 0.42. *Proteobacteria*, *Verrucomicrobiota* increased significantly in DKD group (*p*< 0.05), and they decreased after Sac/Val treatment but the differences were not significant (*p*> 0.05). *Euryarchaeota*, *Synergistata* and *Fusobacteriota* were increased in fecal samples of DKD mice (**Figure 3A**).

At the genus level, the abundance of *Bacteroides* increased in DKD group and En group, whereas the *Muribaculaceae* and *Muribaculum* decreased in both the two groups compared with Con group. The abundance of genus Rikenellaceae_RC9_gut_group was highest in En group. In addition, *Escherichia-Shigella*, *Akkermansia*, *Faecalibacterium*, *Erysipelotrichaceae_UCG-003*, were enriched in DKD group, but the latter two decreased after Sac/Val treatment. On the contrary, *Dubosiella* decreased significantly in DKD group, but tended to increase after Sac/Val treatment. Genus *Paraprevotella*, *Oscillospiraceae_UCG-002*, *Agathobacter* and *Methanobrevibacter* were only enriched in DKD group (**Figure 3B**).

Using the LefSe analysis, we identified the key biomarkers in each group at the genus level, that is, the bacterial genera with significant differences between the groups. According to the criteria of LDA score greater than 2.5 and *p*< 0.05, 13 key biomarkers were identified in Con group, including *Muribaculaceae*, *Alistipes*, *Muribaculum*, *Dubosiella* and several other genera. Meanwhile, 33 and 21 marker genera were identified in DKD group and En group respectively. In DKD mice, the biomarkers mainly included *Prevotella*, *Escherichia_Shigella*, *Christensenellaceae_R_7_group* and *Akkermansia*. For the mice of En group, they were *Bacteroides*, *Rikenellaceae_RC9_gut_group*, *Parabacteroides*, *Lactobacillus*, *Erysipelatoclostridium* and so on (**Figure 3C**). On the whole, Sac/Val treatment altered the composition of gut microbiota.

### Prediction of gut microbiome function in different groups of mice

**Figure 4:**
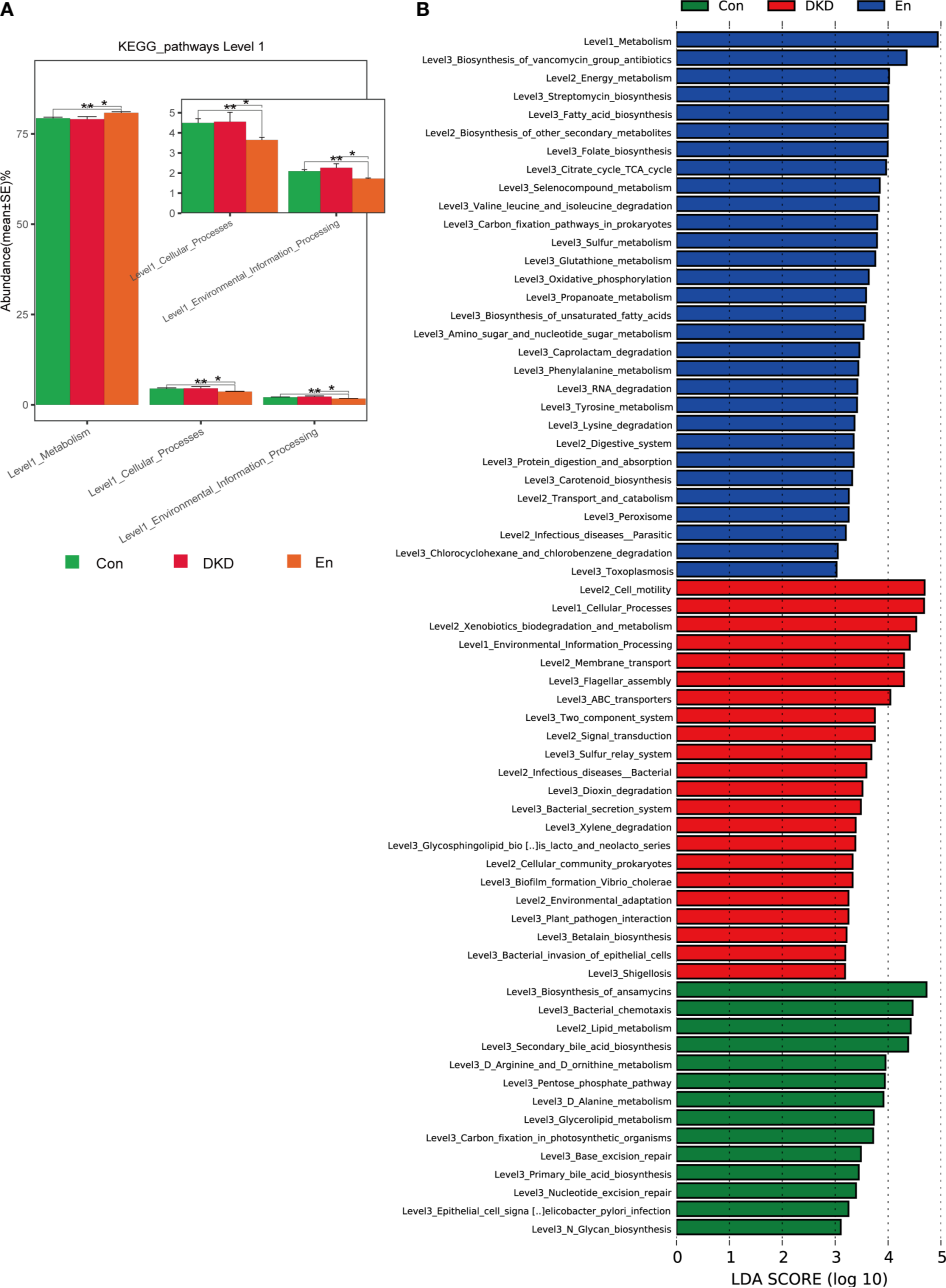
Predictive analysis of gut microbiota function based on KEGG database. **(A)** Complex bar plot of KEGG pathway abundance comparisons at level 1 among groups *p < 0.05, **p < 0.01. **(B)** LefSe analysis of KEGG pathways (LDA ≥ 3).

We also conducted a functional prediction analysis of gut microbiome. At the first level of KEGG pathways, metabolic pathways can be grouped into 6 categories. According to the predictive results, an increase of metabolism was observed in En group compared with the other two groups (*p*< 0.05). Meanwhile, cellular process and environmental information process were decreased in En group (*p*< 0.05). Although environmental information process in DKD group increased, it was not statistically significant (*p*> 0.05) (**Figure 4A**). Consistently, through LefSe analysis, we found that in Con group and En group, metabolism was very active. Differently, the relatively active pathways in the DKD group were cellular process, environmental information process and human diseases, including bacterial invasion of epithelial cells and shigellosis (**Figure 4B**). These results suggested that Sac/Val treatment modulates DKD bacterial composition and its predicted function also differs from the DKD group.

### Differences in the plasma metabolome among the three groups of mice

**Figure 5:**
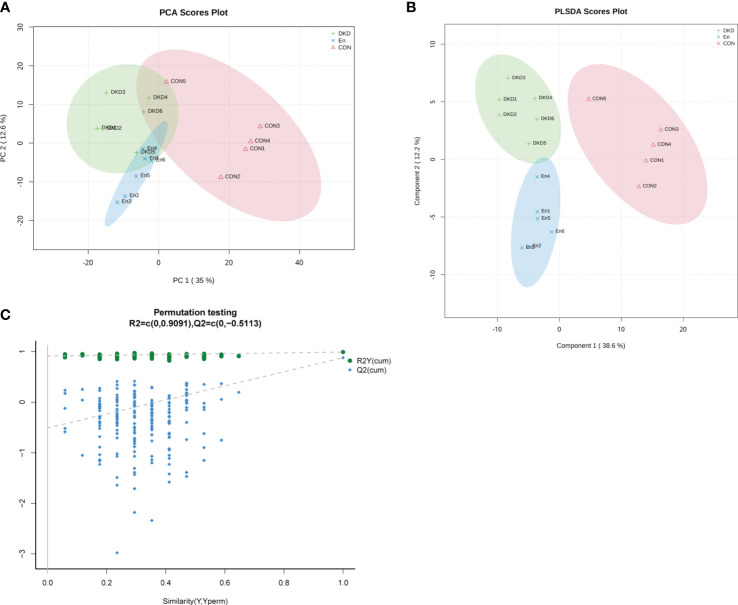
Characteristics of metabolic profiles in each group. **(A)** PCA scores plot. **(B)** PLS-DA scores plot. Component 1: explainability of the first principal component; Component 2: explainability of the second principal component. **(C)** Permutation testing plot.

We performed plasma metabolomics analysis on three groups of mice. A total of 276 cationic and 372 anionic metabolites were identified. The PCA analysis revealed that the metabolic spectrum of DKD and En groups were significantly different from that of Con group. Although it is not very obvious, there was a trend of separation between DKD and En groups. The accumulative R^2^X was 0.58, which indicated that the effect of the model was good (**Figure 5A**). The PLS-DA scores plot showed the plasma metabolic profiles of the three groups of mice were separated from each other (accumulative R^2^X = 0.706, accumulative R^2^Y = 0.99, accumulative Q^2^ = 0.877, **Figure 5B**). Permutation testing showed the Q^2^ value of all newly constructed PLS-DA models was lower than the original model, and the Q^2^ intercept was lower than 0, suggesting our present PLS-DA model has the best fitting effect (**Figure 5C**). Guided by above results, we concluded that the metabolome of En group mice was different from the other two groups to some extent.

### Cluster analysis of differential metabolites

**Figure 6:**
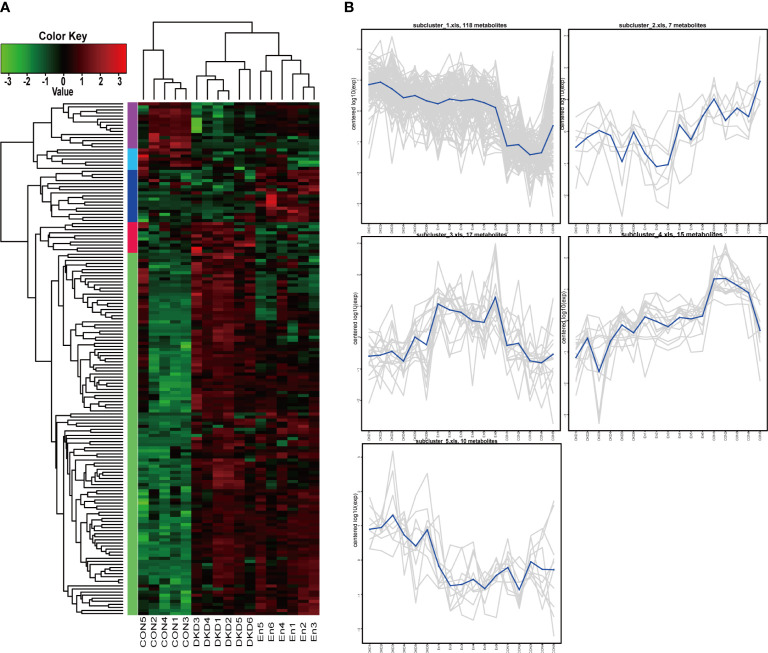
Cluster analysis of differential metabolites. **(A)** Heat map of differential metabolite cluster. **(B)** Line plots of expression trend of 5 subclusters.

In total, 167 important different metabolites among groups were screened. Among which, 53 were positive ionization modes, 114 were negative ionization modes, the expression levels of these metabolites were displayed in the form of heat maps (**Figure 6A**). We found that the metabolic profile of DKD mice was significantly different from that of Con mice. Sac/Val treatment only reversed the abnormal plasma level of some metabolites. At the same time, we performed cluster analysis of their expression patterns. They can be clustered into 5 clusters. Cluster1 and cluster 5 contained 118 and 10 differential metabolites respectively, Cluster 3 containing 17 differential metabolites, had higher expression in En group (**Figure 6B**).

### Analysis of metabolic pathways

**Figure 7:**
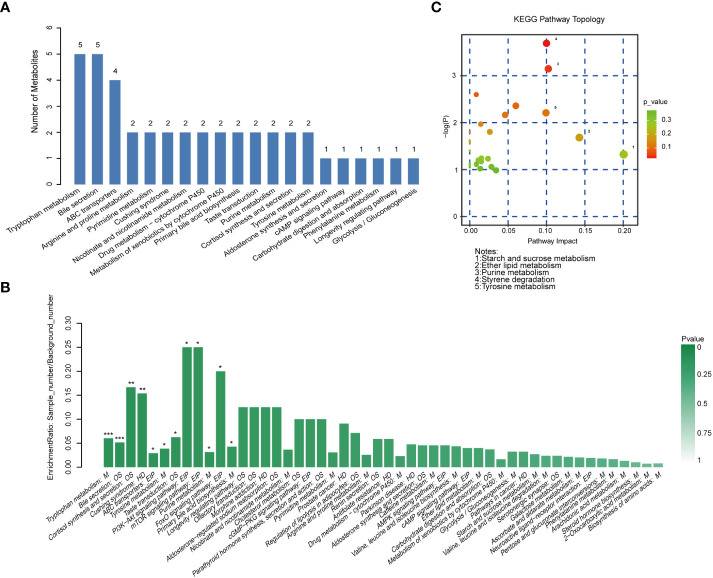
Metabolic pathway analysis. **(A)** Histogram of the top 20 pathways containing the most different metabolites. **(B)** Histogram of KEGG pathway enrichment analysis of differential metabolites. ***p < 0.001, **p < 0.01, *p < 0.05. **(C)** Bubble chart of KEGG topology analysis.

After obtaining the ID number of the KEGG of the differential metabolites, we performed pathway annotation analysis on the differential metabolites through the KEGG database. Among the top 20 pathways containing the most differential metabolites, the top two were tryptophan metabolism and bile secretion, they both contained 5 differential metabolites, followed by ABC transporters containing 4 differential metabolites, and then it was Arginine and proline metabolism, Pyrimidine metabolism and so on (**Figure 7A**). KEGG pathway enrichment analysis of differential metabolites showed that tryptophan metabolism: M and bile secretion: OS had the highest significance of enrichment (*p*< 0.001). The two metabolic pathways PI3K−Akt signaling pathway: EIP and mTOR signaling pathway: EIP have the highest enrichment rate (*p*< 0.05). In addition, the enrichment of cortisol synthesis and secretion, tyrosine metabolism, purine metabolism and primary bile acid biosynthesis pathways were also significant (**Figure 7B**). For these different metabolites, we further conducted a MetPA analysis to evaluate the relative magnitude of their influence on the pathways. The important different metabolites involved in styrene degradation (*p*< 0.05, FDR = 0.337, impact value = 0.100) and purine metabolism (*p*< 0.05, FDR = 0.387, impact value = 0.102) pathways have significant effects on their respective pathways (**Figure 7C**).

**Figure 8:**
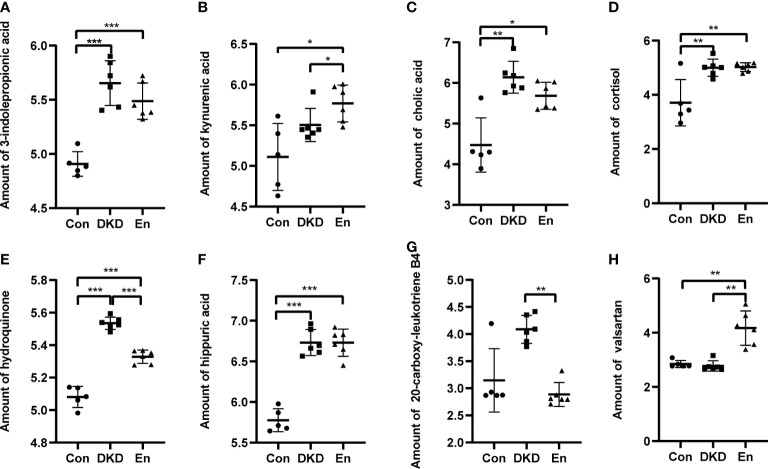
Relative amount of some differential metabolites. **(A)** 3-indolepropionic acid. **(B)** Kynurenic acid. **(C)** Cholic acid. **(D)** Cortisol. **(E)** Hydroquinone. **(F)** Hippuric acid. **(G)** 20-carboxy-leukotriene B4. **(H)** Valsartan. *p < 0.05, **p < 0.01, ***p < 0.0001.

Guided by these results, we screened several metabolites of concern from 167 metabolites of important difference. And we explored their expression characteristics. The metabolites 3-indolepropionic acid and kynurenic acid of tryptophan metabolism pathway are enriched in DKD and En groups (**Figures 8A, B**). Cholic acid (CA) belongs to both the bill secret pathway and the primary bill acid biosynthesis pathways, whose expression was the highest in DKD group. Although the pasma level of cholic acid were decreased in En group, it was still higher than that in Con group (**Figure 8C**). The cortisol expression in both DKD and En groups was higher than that in Con group (**Figure 8D**). Hydroquinone is one of the metabolites of tyrosine, and it was enriched in DKD group (**Figure 8E**). Hippuric acid is uremic toxin, with increased expression in DKD group, but no significant decrease after Sac/Val treatment (**Figure 8F**). Leukotriene B4 (LTB4) can be metabolized to 20-carboxy-leukotriene B4 in leukocytes and hepatocytes. 20-Carboxy-leukotriene B4 increased in DKD group and could be reversed by Sac/Val treatment (**Figure 8G**). Finally, as the main components of Sac/Val, valsartan was the highest in the mice of En group, which is in line with our expectation (**Figure 8H**).

## Discussion

Sac/Val is a new drug mainly used to treat heart failure through blocking both RAAS and neprilysin pathways (24). According to reports, Sac/Val had a protective effect on heart and kidney damage in HPD-feeding CRS rat (19, 25), and it could suppress fibrosis *via* ERK signaling pathway and PKG activity in rats (26). Therefore, the benefits of Sac/Val therapy may not be limited to improving heart failure.

In this study, the application of Sac/Val had no significant effect on RBG of DKD mice, which conformed to previous study regarding the animal (27). As documented in this literature, early or late use of valsartan or Sac/Val cannot improve glucose utilization in diabetic rats. However, other research differently stated that Sac/Val treatment was helpful for controlling the blood glucose in hypertensive patients with HFrEF (18). Interestingly, a recent study found that the protease neprilysin can cleave glucagon into five inactive fragments *in vitro*. Sacubitril is an inhibitor of the protease neprilysin, and treatment with Sac/Val could trigger the hyperglucagonemia and an increase in postprandial amino acid catabolism (28). Further research can explain these mixed results.

In preclinical study of kidney injury, Sac/Val could reduce serum creatinine, counter hypoxia and oxidative stress, suppress proinflammatory cytokines, and inhibit fibrosis (29). The Sac/Val treatment reduced the creatinine level at the end of the experiment, this further corroborates previous report (29). The pity is that most of the current studies on the effect of Sac/Val on the improvement of renal function are carried out in patients combined with heart failure (30). More clinical experimental evidence in patients with diabetes nephropathy even other kidney diseases is needed in the future.

Next, Sac/Val was found to be able to improve the kidney damage of DKD mice. In short, Sac/Val treatment can significantly attenuate renal fibrosis, inflammatory cell infiltration, and glycogen deposition. EndMT and renal fibrosis are the key links of DKD progression (31). We further verified that compared with DKD group, Sac/Val reduced the expression of vimentin, collagen IV, and fibronectin, which were marker proteins of EndMT and renal fibrosis. Meanwhile, Sac/Val restored the expression of nephrin, an podocyte phenotype protein. Consistently, Sac/Val was predicted to decrease collagen expression and anti-fibrosis through either ERK overexpression or PKG knockdown, both of which decreased Smad3 activity (26). Recently, scholars found that reduced circulating markers of inflammation and fibrosis correlated with improved hemodynamic during chronic treatment with Sac/Val, and Sac/Val had the anti-fibrotic and anti-inflammatory effects on the myocardium of HFrEF patients (32). Jointly, these results showed that Sac/Val was central to kidney protection in DKD.

Moreover, to understand whether the renal benefits of DKD were related with changes in the gut microbiota, we further sequenced and analyzed the gut microbiota in each group. An increased β-diversity of gut microbiota was observed in DKD, while it was similar with normal mice in Sac/Val treated mice. In present study, the microbial community composition of DKD was different from that of the other two groups, which was similar to previous report (33). Similar to previous studies, we found an obvious increase in the ratio of *Firmicutes* to *Bacteroidota* in DKD mice compared with the control mice (34). This change was reversed by the application of Sac/Val. Some studies indicated that the ratio of *Firmicutes* to *Bacteroidota* was a biomarker of microbiome dysbiosis associated with obesity, but it was still controversial (35). At the genus level, *Muribaculaceae* was the most abundant genus in the three groups. Such genus was a biomarker of Con mice, but its abundance decreased significantly in mice of DKD and En groups. *Muribaculaceae* was the butyrate-producing bacteria, which was correlated with the formation of colonic inner mucus layer and restoration of intestinal mucosal barrier function (36). Therefore, the reduction of *Muribaculaceae* abundance in DKD state may increase intestinal permeability and the risk of bacterial translocation or harmful bacterial metabolites entering the circulation.

*Escherichia-Shigella* belongs to *Enterobacteriaceae*. Our results showed that *Escherichia-Shigella* was enriched in the gut microbiota, playing the role of biomarker of DKD mice. This was consistent with previous research (36, 37). *Escherichia-Shigella*, as a kind of Gram-negative bacilli, can produce an endotoxin-lipopolysaccharide (LPS) (38). It can also directly destroy intestinal tight junction proteins and gut barrier function (36, 38). Consequently more LPS will enter the blood from the “leaky intestine”, which caused chronic systemic inflammation and disturbed glucose metabolism (38). Indeed, *Escherichia-Shigella* was found to be positively correlated with BMI in human-related study (37). Aligned with the enrichment of *Escherichia-Shigella* in DKD group, the prediction results of bacterial function showed that Shigellosis and Bacterial invasion of epithelial cells were also enriched in DKD group. Ideally, after the treatment with Sac/Val, this situation was reversed.

It was surprising to find that *Akkermansia* was enriched in DKD model group. Indeed, this genus has been widely reported as a beneficial microbiota (39), whose colonization can improve gut barrier and metabolic disorders, such as glucose intolerance, insulin resistance, and obesity (40). Therefore, scholars can explain this inconsistency in further study. *Faecalibacterium* and *Erysipelotrichaceae_UCG-003* were enriched in DKD mice. They were reported to have positive relationship with homeostatic model assessment of insulin resistance (41). It is worth noting that *Erysipelotrichaceae UCG-003* is capable of inducing Th17 cells in IBD patients (42). Similarly, *Erysipelotrichaceae* was found to be enriched in individuals suffering from inflammatory diseases and obesity (41). The abundance of the above two harmful bacteria was extremely low in the Con group and in the mice receiving Sac/Val treatment. *Lactobacillus*, *Bacteroides*, *Parabacteroides* and *Rikenellaceae_RC9_gut_group* were biomarkers of En group, and they were most enriched in the Sac/Val treatment group. Transplantation of *Lactobacillus* can reduce inflammatory response. *Lactobacillus* and *Parabacteroides* were bacteria that can produce short-chain fatty acids (SCFAs) (43). SCFAs can enhance the autophagy of renal tubular cells and attenuate the renal fibrosis in diabetic mice (44). *Bacteroides* is also believed to produce SCFAs by degrading cellulose in food, which can improve some metabolic diseases, such as diabetes and obesity, and inhibit the inflammatory responses of renal tissue in DKD (45). *Rikenellaceae_RC9_gut_group* were negatively correlated with serum lipids parameters, glucose and insulin (46, 47), and this bacterium may play a role in the metabolism of glucose and lipid (48). Briefly, these results demonstrated that the intervention of Sac/Val partially reshaped the composition of gut microbiota, reduced the abundance of some harmful bacteria and increased the abundance of beneficial bacteria, especially the SCFAs-producing bacteria. This may be one of the mechanisms that Sac/Val therapy can reduce renal fibrosis. In addition, through functional prediction analysis, we verified that Sac/Val not only reshaped the structure of intestinal flora of DKD, but also improved the function of gut microbiome.

Finally, we explored the underlying mechanism of Sac/Val ameliorating renal damage from the aspect of plasma metabolomics. Tryptophan metabolism was more active in DKD and En groups, which further validates previous reports (23, 49). Disturbed tryptophan metabolism was related to the decrease in eGFR (49). Tryptophan can produce kynurenine, which can be further metabolized into kynurenic acid (Kyna) and other substances (50). Kyna was increased in T2D and positively associated with T2D occurrence (50). In the current study, although the level of Kyna in DKD group had an upward trend, it did not reach statistical significance. However, it was higher in En group, indicating that Sac/Val does not improve renal damage by regulating Kyna metabolism. Plasma CA concentrations increased in both DKD and En groups. CA promoted intestinal absorption of lipids, especially cholesterol (51). Therefore, we speculated that the increase of CA was related to the diabetic status and high-fat diet in the mice of both the two groups. The CA was negatively associated with insulin sensitivity in healthy adults, obese and T2D patients (52). However, another study stated that CA treatment activated the farnesoid X receptor (FXR), and significantly attenuated the increased mRNA expression of fibronectin and α-SMA in the kidneys of db/db mice, and improve renal pathological damage (53). However, the research on diabetic nephropathy and CA has rarely been conducted..

The cortisol was accumulated in mice of both DKD and En groups. Previous study also showed that plasma levels of cortisol increased in the diabetic state (54). Cortisol stimulation caused an increased expression of pro-fibrotic genes in human renal mesangial cells in DKD (55). Hippuric acid, as a uremic toxin produced by dietary polyphenols metabolism under the action of gut microbiota, increased in DKD condition. It could promote renal fibrosis in chronic kidney disease (56). Sac/Val intervention did not change the plasma level of hippuric acid. LTB4 increased in type 1 diabetes and mediated the systemic inflammation and macrophage reprogramming. LTB4/LTB4 receptor (BLT1) can induce fat loss and increased macrophage lipid uptake (57). LTB4 is metabolized into 20-hydroxy-leukotriene B4 and 20-carboxyl-leukotriene B4 in leukocytes and hepatocytes. In this study, 20-carboxy-leukotriene B4 was greatly increased in DKD mice, but reversed by Sac/Val. The 20-carboxy-leukotriene B4 may reflect the production of leukotrienes. To our knowledge, few studies have focused on the 20-carboxy-leukotriene B4. This is also consistent with the reduction in pro-inflammatory bacteria in the gut microbiota previously mentioned in this paper. Jointly, we argued that that Sac/Val treatment only slightly changed the plasma metabolic spectrum of DKD.

However, there are several limitations in this study. This is a preliminary study in mice, and has not been verified by clinical experiments. Moreover, this study did not compare the difference between Sac/Val and valsartan in their effects on gut microbiota and kidney damage of DKD. Future research is needed to make up for these deficiencies.

In conclusion, we found that the abundance of harmful bacteria in DKD increased. The Sac/Val therapy led to the decrease of pathogenic bacteria and expansion of beneficial gut bacteria, as well as the improvement of renal fibrosis and function in DKD. Sac/Val may play a renal protective role in DKD. It is necessary to carry out further verification in clinical experiments.

## Data availability statement

The datasets presented in this study can be found in online repositories. The links and accession numbers are as follows: https://submit.ncbi.nlm.nih.gov, PRJNA876080; https://www.ebi.ac.uk/metabolights, MTBLS6040 and MTBLS6377. This study is a branch of our overall project, and the Con and DKD groups also appears in another necessary sub study (58).

## Ethics statement

The animal study was reviewed and approved by the Ethical Committee of Experimental Animal Care of First Affiliated Hospital of Zhengzhou University.

## Author contributions

We acknowledge all authors who have contributed to this work. Authors ZZ and JS designed and funded this study. Author PW wrote the manuscript and completed the experiments with the help of RG and HL. Authors XB, WC and YZ conducted the data analysis. All authors contributed to the article and approved the submitted version.

## Funding

This study was funded by the National Natural Science Foundation of China (Grant Nos.81873611 and 82170738).

## Conflict of interest

The authors declare that the research was conducted in the absence of any commercial or financial relationships that could be construed as a potential conflict of interest.

## Publisher’s note

All claims expressed in this article are solely those of the authors and do not necessarily represent those of their affiliated organizations, or those of the publisher, the editors and the reviewers. Any product that may be evaluated in this article, or claim that may be made by its manufacturer, is not guaranteed or endorsed by the publisher.
